# Work engagement and associated factors among healthcare professionals in the post-pandemic era: a cross-sectional study

**DOI:** 10.3389/fpubh.2023.1173117

**Published:** 2023-07-27

**Authors:** Yiya Wang, Li Tang, Lezhi Li

**Affiliations:** Clinical Nursing Teaching and Research Section, The Second Xiangya Hospital, Central South University, Changsha, Hunan, China

**Keywords:** work engagement, healthcare professionals, post-pandemic era, factors, cross-sectional study

## Abstract

**Background:**

With the shift of strategy in fighting COVID-19, the post-pandemic era is approaching. However, the “hard times” for healthcare systems worldwide are not yet ending. Healthcare professionals suffer negative impacts caused by the epidemic, which may seriously threaten their work motivation, concentration, and patient safety.

**Objective:**

Investigating the status and factors associated with Chinese healthcare professionals’ work engagement in the post-pandemic era.

**Methods:**

A cross-sectional study was conducted to investigate healthcare professionals from 10 hospitals in Hunan Province. Data were collected using demographic characteristics, Generalized Anxiety Disorder-2, Patient Heath Qstionaire-2, Utrecht Work Engagement Scale, Work-Related Basic Need Satisfaction Scale, National Aeronautics and Space Administration-Task Load Index, and self-compassion scale. Descriptive and multiple linear regression analyses explored the factors associated with work engagement.

**Results:**

A total of 1,037 eligible healthcare professionals participated in this study, including 46.4% of physicians, 47.8% of nurses, and 5.8% of others. The total mean score of work engagement was 3.36 ± 1.14. The main predictor variables of work engagement were gender (*p* = 0.007), years of work experience (*p* < 0.001), whether currently suffering challenges in the care of patients with COVID-19 (*p* = 0.003), depression (*p* < 0.001), work-related basic need satisfaction (*p* < 0.001), and mindfulness (*p* < 0.001).

**Conclusion:**

Healthcare professionals have a medium level of work engagement. Managers need to pay attention to the physical and psychological health of healthcare professionals, provide adequate support, help them overcome challenges, and acknowledge their contribution and value to improve their work engagement, enhance the quality of care and ensure patient safety.

## 1. Introduction

Since December 2019, the immediate global pandemic of COVID-19 has brought enormous challenges and shocks to the world’s healthcare systems. In order to respond effectively to this public healthcare event, worldwide healthcare professionals, including doctors, nurses, and other disciplines, have been fully engaged in the care of patients with COVID-19 and the prevention and control of the epidemic. However, the enormous number of patients with COVID-19 and the high risk of infection further strain the available medical resources and the medical environment. Frontline healthcare professionals caring for COVID-19 patients are under heavy workloads and psychological stress (1). Healthcare professionals’ physical and psychological conditions have become critical research fields during the pandemic outbreak. Studies showed that healthcare professionals suffered from a range of trouble, such as anxiety, depression, burnout, and insomnia during the outbreak (2–4). These issues are detrimental to healthcare professionals’ physical and psychological health, seriously affect the quality of care, and threaten patient safety.

With the shift of policy and strategy in fighting COVID-19, global epidemic control has gradually been liberalized. The deregulation of the policy and the downgrading of prevention and control levels have brought tremendous pressure and challenges to healthcare professionals, including the surge of infections, especially among healthcare professionals, marked increased workload, and the shortage of medical resources. Significant deterioration in the quality of care, working conditions, occupational health, and patient safety compared to the situation before the COVID-19 outbreak (5). Effective medicines are still lacking, and the battle against COVID-19 is ongoing. As a professional group, healthcare professionals have an essential role in the care of infected patients and in preventing and controlling the pandemic. However, these disadvantages can seriously threaten the work motivation, dedication, efficiency, and quality of healthcare professionals. Retaining skilled healthcare professionals and continuing their engagement is a huge challenge for healthcare systems. Therefore, investigating the work engagement of healthcare professionals in this particular context is vital.

As an essential component of the PERMA (Positive emotion, Engagement, Relationship, Meaning, Accomplishment) model in positive psychology (6), work engagement has become a popular research topic in positive organizational behavior and human resource management. Work engagement is a work-related positive, enriching emotional and cognitive status comprised of vigor (i.e., high levels of psychological energy during work), dedication (i.e., a sense of significance, enthusiasm and challenge with regard to work), and absorption (i.e., total immersion in one’s work) (7). Studies demonstrated that high work engagement was associated with low burnout, reduced turnover intention, increased job satisfaction, enhanced work performance and care quality, promoted patient health outcomes, and positively impacted healthcare systems (8–12). Cai et al. (13) investigated the work engagement of Chinese nurses before the COVID-19 outbreak (2019) and showed that nurses’ work engagement was at a medium level. Yin et al. (14) examined the status and typology of frontline nurses’ work engagement in China at the beginning of the pandemic (2020) and found that more than 40% of nurses’ work engagement was low. However, another study from Spain indicated a high level of work engagement among healthcare professionals at the beginning of the pandemic (15). Wijngaards et al. (16) discovered that frontline healthcare professionals’ work engagement was slightly above average during a period when the Netherlands was gradually relaxing the COVID-19 protective measures (2020). From this, variability in the level of work engagement of healthcare professionals between different studies and contexts exists. Up to now, the COVID-19 epidemic has been lasting for more than 3 years. Confronted with a liberalized epidemic policy, the number of infected patients has soared. Both the healthcare workforce and resources have been challenged and shocked to some extent. In the ongoing battle against COVID-19, healthcare professionals are mentally tensed, exhausted, and also have to deal with their own and family members’ infections, which will make them overloaded. Therefore, in this particular context, these negative effects may still be detrimental to the work engagement of healthcare professionals. Notably, no relevant research is available on the status and influencing factors of healthcare professionals’ work engagement in the post-epidemic era, namely after the epidemic liberalization.

**Figure 1:**
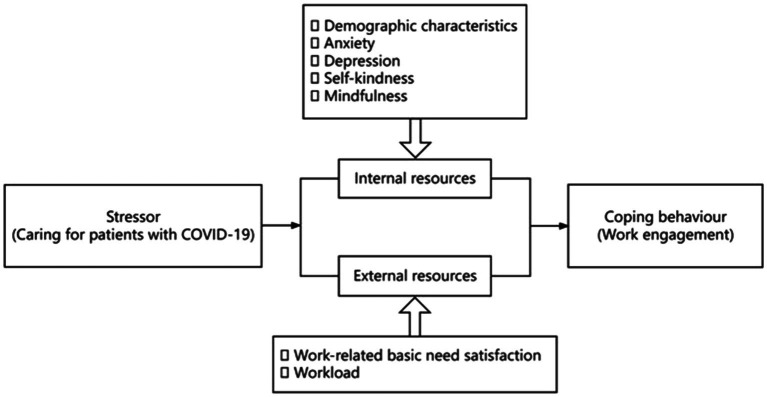
The framework in this study (self-designed based on COR and research hypothesis).

The conservation of resource theory (COR) was developed by Hobfoll in 1989, which explained stress and burnout in terms of the loss and gain of resources and stated that individuals always strove to protect, maintain and acquire valuable resources (17, 18). This theory suggests that the resources of individuals are limited, including energy, time, and emotions. The individual’s resources may be depleted when they are exposed to stressors such as stressful work, role conflict, etc., and a variety of negative outcomes may occur. Accordingly, this study regarded work engagement as a coping behavior of healthcare professionals to protect resources in stressful situations and investigated the potential factors associated with work engagement of healthcare professionals from internal and external resources (see Figure 1) to provide suggestions for improving the work engagement of healthcare professionals.

## 2. Materials and methods

### 2.1. Design and setting

A cross-sectional study was conducted to investigate the work engagement of healthcare professionals. Data were collected in 10 hospitals from Hunan Province in January 2023 (after adjusting epidemic prevention and control policies in China). This study followed the STROBE reporting guidelines.

### 2.2. Participants

Purposive sampling was used to recruit healthcare professionals (doctors, nurses, and others) in 10 hospitals from the Hunan Health Management Association (HNHMA). Inclusion criteria: (i) having professional qualifications; (ii) currently working on the clinical frontline, with no limitation on the departments; and (iii) willing to participate in this study. Exclusion criteria: (i) training; (ii) internship; and (iii) rotation of departments. The sample size for linear regression is at least 10 times the number of independent variables (19). Assuming that all independent variables enter into the regression equation, the number of independent variables for this study is 41 (including dummy variables) and the required sample size is at least 451, considering the 10% invalid questionnaire. Finally, a total of 1,037 healthcare professionals were enrolled and met this requirement.

### 2.3. Instruments

#### 2.3.1. Demographic characteristics

The basic information of participants, including gender, age, education level, marital status, occupation, professional title, hospital grade, department, years of work experience, the status of COVID-19 infection, the severity of caring for patients with COVID-19, whether having experience in the care of patients with COVID-19, and whether currently suffering challenges in the care of patients with COVID-19 and the type of challenges encountered.

#### 2.3.2. Generalized anxiety disorder-2

The Generalized Anxiety Disorder-2 (GAD-2) was used to assess participants’ anxiety symptoms in the past 2 weeks (20). The scale consists of two items. Each item is scored on a 4-point scale. The total score ranges from 0 to 6, with higher scores indicating higher anxiety levels. The cut-off score is 3. The scale has been used to screen anxiety in healthcare professionals (21). The Cronbach’s α coefficient in this study is 0.845.

#### 2.3.3. Patient health questionnaire-2

The Patient Health Questionnaire-2 (PHQ-2) was used to measure participants’ depression symptoms in the past 2 weeks (22). The scale consists of two items. Each item is scored on a 4-point scale. The total score ranges from 0 to 6, with higher scores indicating higher depression levels. The cut-off score is 3. The scale has been used to screen depression in healthcare professionals (21). The Cronbach’s α coefficient in this study is 0.882.

#### 2.3.4. Utrecht work engagement scale

The Utrecht Work Engagement Scale (UWES-9) was developed by Schaufeli et al. (23) to measure healthcare professionals’ work engagement. The UWES-9 comprises nine items clustered in three dimensions (vigor, dedication, and absorption), using a 7-point scale (from 0 = never to 6 = always). The total mean score ranges from 0 to 6 scores. A higher score suggests greater work engagement. The Cronbach’s α coefficient in this study is 0.944.

#### 2.3.5. Work-related basic need satisfaction scale

The Work-Related Basic Need Satisfaction Scale (W-BNS) was developed by Van den Broeck et al. (24) to measure healthcare professionals’ work-related basic need satisfaction. The W-BNS scale includes 18 items and divides into three dimensions (relatedness, competence, and autonomy), rating on a five-point scale (from 1 = completely disagree to 5 = completely agree). The Cronbach’s α coefficient in this study is 0.870.

#### 2.3.6. National Aeronautics and Space Administration-task load index

The National Aeronautics and Space Administration-Task Load Index (NASA-TLX) was developed by Hart et al. (25) to measure healthcare professionals’ workloads. The NASA-TLX consists of 6 items that evaluate six dimensions regarding workload, including mental demand, physical demand, temporal demand, performance, effort, and frustration. The score of each item ranges from 0 (low load) to 20 (high load). The lower the performance score, the more perfect the self-performance and the lower the workload. The total score is the sum of each item’ score, ranging from 0 to 120, with higher scores indicating a higher load (26). The Cronbach’s α coefficient in this study is 0.813.

#### 2.3.7. Self-compassion scale

The self-compassion scale was developed by Neff et al. (27), with six subscales (self-kindness, common humanity, mindfulness, self-judgment, isolation, and overidentified). The elements of self-compassion are distinct and can be measured separately (28). This study used the subscales of self-kindness and mindfulness separately to measure health professionals’ self-kindness and mindfulness, respectively. The Cronbach’s α coefficient for the subscale of self-kindness in this study is 0.868, and the subscale of self-kindness is 0.893.

### 2.4. Data collection

This study used electronic questionnaires to collect data via Wenjuanxing.^1^ The front page of the electronic questionnaire was the information statement, including the study overview and data confidentiality pledge. Questions can only be entered if the participant clicks to agree to participate in this study, otherwise, they will be automatically logged out. Each ID was set to be filled in only once, and each question was compulsory. In order to ensure the integrity of the data, the questionnaire will be submitted only after all the questions have been completed. Before the survey, the research team obtained informed consent from the hospitals. Then, the researchers explained the purpose of the study, the subjects, and the precautions for department chiefs and nurse managers and sent the QR code of the questionnaire to them via WeChat. Finally, the department chiefs and nurse managers used the uniform information template created by the researcher (including the purpose, significance, subjects, and instructions) to introduce this study to healthcare professionals and motivated healthcare professionals who met the criteria to fill it out carefully in the workgroup. After the electronic questionnaires were completed, two trained researchers checked each questionnaire to ensure their quality.

### 2.5. Data analysis

Data were analyzed by IBM SPSS 26.0. The scores of work engagement, work-related basic need satisfaction, workloads, self-kindness, and mindfulness showed approximately normal distributions (checked by histograms and normal curves). Mean, standard deviations, frequency, and percentage were used to describe variables. One-way analysis of variance (ANOVA) and *t*-tests were used to examine the influence of different independent variables on work engagement. Pearson correlation was used to identify the relationships between work-related basic need satisfaction, workloads, self-kindness, and mindfulness with work engagement. Significant variables were included in multiple linear regression for further analysis. Dummy variables of unordered multi-categorical variables used the “Enter” method, while others used the “Stepwise” method to select. The values of “alpha to enter” and “alpha to remove” were, respectively, 0.05 and 0.10. A two-tailed *p*-value under 0.05 was considered statistically significant.

### 2.6. Ethical approval

This research was approved by the Ethics Committee of the Second Xiangya Hospital, Central South University (XGFYYJHL-2020). The data collected was encrypted and available only to the researchers.

## 3. Results

### 3.1. Characteristics of participants

A total of 1,037 eligible healthcare professionals participated in this study, including 46.4% of physicians, 47.8% of nurses, and 5.8% of others. Most of the participants were 25 years and older. The majority had 11–15 years of experience (23.8%). Over 90% were infected with COVID-19. A large proportion had experience caring for patients with COVID-19 (65.0%). Less than 1/3 had anxiety (31.9%) and depression (24.8%). Detailed information was shown in Table 1. In addition, over two-thirds suffered challenges in the care of patients with COVID-19. The most common challenges were insufficient workforce and larger workloads than before. Details were presented in Table 2.

**Table 1:** Characteristics of participants and results of one-way ANOVA and *t*-test (*N* = 1,037).

Variables	*N*	%	Work engagement (Mean ± SD)	t/F	*P*
Gender				5.027	<0.001
Male	301	29.0	3.64 ± 1.18		
Female	736	71.0	3.25 ± 1.10		
Age (year)				13.264	<0.001
<25	76	7.3	3.29 ± 1.07		
25 ~ 35	505	48.7	3.17 ± 1.12		
36 ~ 45	317	30.6	3.45 ± 1.11		
46 ~ 55	125	12.1	3.90 ± 1.11		
>55	14	1.4	4.10 ± 0.72		
Education level				0.298	0.827
College degree or lower	166	16.0	3.35 ± 1.22		
Undergraduate degree	771	74.3	3.36 ± 1.13		
Master’s degree	90	8.7	3.39 ± 1.08		
Doctor’s degree	10	1.0	3.69 ± 0.63		
Marital status				11.178	<0.001
Unmarried	210	20.3	3.04 ± 1.11		
Married	804	77.5	3.44 ± 1.13		
Others	23	2.2	3.69 ± 0.97		
Occupation				15.167	<0.001
Physician	481	46.4	3.52 ± 1.16		
Nurse	496	47.8	3.17 ± 1.09		
Others	60	5.8	3.69 ± 1.01		
Professional title				9.651	<0.001
Primary title	410	39.5	3.24 ± 1.18		
Intermediate title	419	40.4	3.34 ± 1.11		
Senior title	208	20.1	3.66 ± 1.04		
Hospital grade				1.928	0.146
Primary	57	5.5	3.35 ± 1.33		
Secondary	308	29.7	3.26 ± 1.15		
Tertiary	672	64.8	3.41 ± 1.11		
Department				1.731	0.069
Emergency	75	7.2	3.13 ± 1.11		
Outpatient	160	15.4	3.60 ± 1.09		
General internal medicine	222	21.4	3.24 ± 1.25		
General surgery	222	21.4	3.42 ± 1.11		
Infectious diseases	12	1.2	2.98 ± 0.85		
GICU	164	15.8	3.34 ± 1.09		
Specialized ICU	53	5.1	3.40 ± 1.00		
Hemodialysis	24	2.3	3.44 ± 1.25		
Respiratory diseases	40	3.9	3.15 ± 1.24		
Obstetrics and gynecology	35	3.4	3.49 ± 0.98		
Pediatrics	30	2.9	3.49 ± 1.00		
Years of work experience				13.866	<0.001
≤5	223	21.5	3.20 ± 1.15		
6–10	245	23.6	3.15 ± 1.13		
11–15	247	23.8	3.27 ± 1.06		
16–20	111	10.7	3.48 ± 1.07		
≥21	211	20.3	3.83 ± 1.12		
The status of COVID-19 infection				0.517	0.596
Infected but recovery	957	92.3	3.37 ± 1.12		
Positive	17	1.6	3.09 ± 1.20		
Negative	63	6.1	3.38 ± 1.33		
The severity of caring for patients with COVID-19					
Mild				−1.080	0.281
Yes	776	74.8	3.38 ± 1.17		
No	261	25.2	3.30 ± 1.03		
Mild with high-risk factors				0.514	0.608
Yes	599	57.8	3.35 ± 1.16		
No	438	42.2	3.38 ± 1.11		
Sub-severe				0.630	0.529
Yes	411	39.6	3.34 ± 1.16		
No	626	60.4	3.38 ± 1.12		
Serious				2.125	0.034
Yes	412	39.7	3.27 ± 1.12		
No	625	60.3	3.42 ± 1.14		
Critical				2.211	0.027
Yes	345	33.3	3.25 ± 1.09		
No	692	66.7	3.42 ± 1.15		
Whether having experience in the care of patients with COVID-19				2.818	0.005
Yes	674	65.0	3.44 ± 1.15		
No	363	35.0	3.23 ± 1.09		
Whether currently suffering challenges in the care of patients with COVID-19				5.468	<0.001
Yes	938	90.5	3.29 ± 1.10		
No	99	9.5	4.01 ± 1.26		
Anxiety				10.148	<0.001
Negative	706	68.1	3.60 ± 1.04		
Positive	331	31.9	2.86 ± 1.17		
Depression				12.724	<0.001
Negative	780	75.2	3.60 ± 1.02		
Positive	257	24.8	2.64 ± 1.15		

ICU, intensive care unit; GICU, general intensive care unit.

**Table 2:** Types of challenges in the care of patients with COVID-19 (*N* = 1,037).

Category	*N*	%
Lack of experience		
Yes	406	39.2
No	631	60.8
Lack of knowledge		
Yes	248	23.9
No	789	76.1
Lack of skills		
Yes	255	24.6
No	782	75.4
Lack of ability of thinking		
Yes	261	25.2
No	776	74.8
Lack of effective drugs		
Yes	457	44.1
No	580	55.9
larger workloads than before		
Yes	595	57.4
No	442	42.6
Length of work longer than before		
Yes	500	48.2
No	537	51.8
Insufficient workforce		
Yes	597	57.6
No	440	42.4
Insufficient equipment		
Yes	408	39.3
No	629	60.7
Shortage of beds		
Yes	340	32.8
No	697	67.2
Patient and family adherence		
Yes	382	36.8
No	655	63.2
Work completion		
Yes	200	19.3
No	837	80.7
Powerlessness		
Yes	397	38.3
No	640	61.7
Others		
Yes	13	1.3
No	1,024	98.7
No challenges		
Yes	99	9.5
No	938	90.5

### 3.2. The scores of work engagement, work-related basic need satisfaction, workload, self-kindness, and mindfulness

As shown in Table 3, The total mean score of work engagement was 3.36 ± 1.14, indicating healthcare professionals had moderate work engagement. The total scores for work-related basic need satisfaction, workload, self-kindness, and mindfulness were 64.90 ± 9.26, 87.58 ± 19.50, 16.47 ± 3.89, and 14.00 ± 3.05, respectively.

**Table 3:** The scores of work engagement, work-related basic need satisfaction, workload, self-kindness, and mindfulness.

Variables	Mean	SD
Work engagement (UWES-9)		
Vigor	3.25	1.15
Dedication	3.53	1.20
Absorption	3.30	1.25
The total mean score	3.36	1.14
Work-related basic need satisfaction (W-BNS)		
Relatedness	22.97	3.69
Competence	23.01	3.70
Autonomy	18.92	3.93
The total score	64.90	9.26
Workload (NASA-TLX)		
Mental demands	14.25	4.36
Physical demands	15.51	4.28
Temporal demands	15.08	4.25
Performance	15.53	3.88
Effort	16.44	3.71
Frustration	10.77	6.21
The total score	87.58	19.50
Self-kindness	16.47	3.89
Mindfulness	14.00	3.05

UWES-9, Utrecht Work Engagement Scale; W-BNS, Work-Related Basic Need Satisfaction Scale; NASA-TLX, National Aeronautics and Space Administration-Task Load Index.

The results of correlation analysis indicated work engagement had a significant positive correlation with work-related basic need satisfaction (*r* = 0.620, *p* < 0.01), self-kindness (*r* = 0.364, *p* < 0.01), and mindfulness (*r* = 0.474, *p* < 0.01), and had a weak negative correlation with workload (*r* = −0.125, *p* < 0.01). Details were presented in Table 4.

**Table 4:** The correlations of work engagement, work-related basic need satisfaction, workload, self-kindness, and mindfulness.

Variables	Work engagement	Work-related basic need satisfaction	Workload	Self-kindness	Mindfulness
Work engagement	1				
Work-related basic need satisfaction	0.620**	1			
Workload	−0.125**	−0.190**	1		
Self-kindness	0.364**	0.352**	−0.115**	1	
Mindfulness	0.474**	0.381**	−0.042**	0.708**	1

**p-values < 0.01.

### 3.3. The relationships between independent variables and work engagement

As indicated in Table 1, variables such as gender, age, marital status, occupation, professional title, years of work experience, whether caring for severe and critical types of patients with COVID-19, whether having experience in the care of patients with COVID-19, whether currently suffering challenges in the care of patients with COVID-19, anxiety, and depression were statistically significant with work engagement (*p* < 0.05). On the contrary, others, such as education level, hospital grade, department, and the status of COVID-19 infection were not statistically significant with work engagement (*p* > 0.05).

### 3.4. The linear regression results among work engagement, work-related basic need satisfaction, workload, self-kindness, mindfulness, and demographic variables

The values of variables entered in the linear regression analyses are detailed in Supplementary Table S1. Results indicated that gender, years of work experience, whether currently suffering challenges in the care of patients with COVID-19, depression, work-related basic need satisfaction, and mindfulness were the significant predictors of work engagement (*R*^2^ = 0.500, adjusted *R*^2^ = 0.495, *F* = 102.436, *p* < 0.001), which explained 50.0% of the variance (see Table 5).

**Table 5:** The result of linear regression analyses.

Variables	*B* (95% CI)	SE	*β*	*P*
Constant	−0.79 (−1.46 to −0.12)	0.34		0.020
Gender	−0.18 (−0.32 to −0.05)	0.07	−0.07	0.007
Years of work experience	0.09 (0.05–0.13)	0.02	0.11	<0.001
Whether currently suffering challenges in the care of patients with COVID-19	−0.26 (−0.44 to −0.09)	0.09	−0.07	0.003
Depression	−0.37 (−0.50 to −0.25)	0.06	−0.14	<0.001
Work-related basic need satisfaction	0.05 (0.05–0.06)	0.00	0.44	<0.001
Mindfulness	0.10 (0.08–0.11)	0.01	0.26	<0.001

*R*^2^ = 0.500, adjusted *R*^2^ = 0.495, *F* = 102.436, *P* < 0.001.

## 4. Discussion

To our knowledge, this study is the first research to investigate healthcare professionals’ work engagement in this post-epidemic era. In our study, the total mean score of healthcare professionals’ work engagement in the post-epidemic era was 3.36 (SD = 1.14), which was moderate overall. This finding was lower than the studies by Gómez-Salgado et al. (15) in the early phases of the epidemic (total mean score = 5.04, SD = 1.14) and Wijngaards et al. (16) in a period of gradually relaxing COVID-19 protective strategies (total mean score = 4.95, SD = 1.02), which suggests that taking measures to improve healthcare professionals’ work engagement after the epidemic liberalization is important. The trajectory of healthcare professionals’ work engagement from pre-epidemic, the early stage of the epidemic, to policy liberalization could be further explored.

Exploring influences is critical to developing measures to improve work engagement. This study found gender, years of work experience, whether currently suffering challenges in the care of patients with COVID-19, depression, work-related basic need satisfaction, and mindfulness are significant predictors of work engagement.

Our study showed that gender significantly affected work engagement. Compared to males, females had relatively lower work engagement. However, Rivera et al. (29) indicated that gender was not statistically significant in work engagement. Possibly due to the following. On the one hand, females were significantly more likely to report negative psychological experiences during the epidemic compared to males (30). On the other hand, in the Chinese traditional cultural context, females are primary caregivers of the family, especially when the families are not well, such as infected with COVID-19, which may take up much energy and lead to less work engagement. The result warrants further validation, given that cross-sectional studies cannot deduce a causal relationship.

Years of work experience had a significant influence on work engagement. The work engagement of healthcare professionals with insufficient work experience was relatively low compared to seniors (more than 5 years of work experience), which was consistent with the findings of Bamford et al. (31). Due to the relative inexperience of younger healthcare professionals, the challenges at work have a higher impact on work engagement than those with more experience. Given this, managers need to value younger healthcare professionals and provide support, especially for novice professionals.

This study found that healthcare professionals faced with challenges caring for patients with COVID-19 were less engaged in the work. In addition, we also discovered that the most common challenges were insufficient workforce and larger workloads than before. The implication is that managers need to provide knowledge and psychological support to help employees cope with the challenges, improve the quality of care and improve their physical and mental health.

Healthcare professionals with high levels of mindfulness had higher work engagement, consistent with Kuang et al. (32). Mindfulness manifests internal resource abundance and has statistical significance on work engagement. Calcagni et al. (33) explored the effects of mindfulness-based interventions on work engagement and demonstrated that the participants’ level of work engagement and performance successfully increased after interventions. Healthcare professionals who screened positive for depression were less engaged in their work. Contrary to mindfulness, depression may be a depleting process of internal resources. Decreased work engagement may act as a proactive coping behavior during resource depletion and increased demands at work (34). Therefore, managers are supposed to boost employees’ mindfulness through training, and promote the positive emotions of healthcare professionals, to enhance work engagement.

This study identified that the higher the satisfaction with work-related basic needs, the higher their work engagement. As a reflection of the abundance of external resources, basic needs satisfaction was significantly associated with work engagement. Cheung et al. (35) found frontline nurses lacked support, especially in psychological aspects, and had low job satisfaction. Luo et al. (36) suggested holistically enhancing the support system and increasing attention and support at the individual, family, and organizational levels. Clinical managers should pay more attention to the needs of employees, provide adequate knowledge, skills and psychological support, acknowledge their contribution and value, and thus increase their work engagement.

Notably, this study found no statistical significance between infection status and healthcare professionals’ work engagement. Possibly a small number of uninfected healthcare professionals and a large number of the infected in this study resulted in a non-significant difference. This result needs further verification. In addition, although a weak negative correlation existed between workload and work engagement, the regression analysis showed that this variable was not a significant contributor to work engagement. The result differed from Wang et al. (37), which showed that workload decreased work engagement among nurses. However, this result was similar to van Mol et al. (38), which suggested that although the relatively high workload in ICUs and a high emotional burden may be an integral part of ICU work, this workload did not affect work engagement. These surveys differed in population, culture, context, and instruments. Given the variation, the results need to be taken seriously. Regardless, our findings further illustrated that despite the unprecedented challenges and burdens faced by the healthcare professionals, their dedication and sense of duty motivated them to fight against the pandemic and build a life-saving defense.

## 5. Implications

In the post-epidemic era, managers and researchers need to focus on healthcare professionals’ physical and psychological health, especially female and young healthcare professionals, provide adequate support (such as training, psychological interventions, adequate medical supplies, etc.) to meet their needs and help them overcome the challenges in caring for patients with COVID-19, acknowledge their contribution and value to increase their work engagement and improve the quality of care. In addition, the findings of this study further validate the COR theory and enrich the application context. The theory may provide significant guidance for future relevant research on work engagement.

## 6. Limitations

Our study also had several limitations. Firstly, the results should be taken cautiously because the sample’s representativeness was limited, and a cross-sectional study cannot deduce causal relationships. Secondly, this study was a preliminary exploration of the influencing factors, possibly overlooking other potential factors. Thirdly, this study only investigated healthcare professionals’ work engagement after the epidemic liberalization and could not describe its changes from the early stage of the epidemic to the liberalization.

## 7. Conclusion

Healthcare professionals had a medium level of work engagement. We also found that gender, years of work experience, whether currently suffering challenges in the care of patients with COVID-19, depression, work-related basic need satisfaction, and mindfulness were significant predictors to work engagement. Managers need to pay attention to healthcare professionals’ physical and psychological health, provide adequate support, help them overcome challenges, and acknowledge their contribution and value to improve their work engagement and enhance the quality of care.

## Data availability statement

The raw data supporting the conclusions of this article will be made available by the authors, without undue reservation.

## Ethics statement

The studies involving human participants were reviewed and approved by Ethics Committee of the Second Xiangya Hospital, Central South University. The patients/participants provided their written informed consent to participate in this study.

## Author contributions

YW: conceptualization, writing – original draft, investigation, methodology, formal analysis, and data curation. LT: conceptualization, investigation, methodology, writing – original draft, and data curation. LL: conceptualization, supervision, review and editing, and data curation. All authors contributed to the article and approved the submitted version.

## Funding

This study was supported by the Central South University Innovation Foundation for Postgraduate, China (2022ZZTS0323).

## Conflict of interest

The authors declare that the research was conducted in the absence of any commercial or financial relationships that could be construed as a potential conflict of interest.

## Publisher’s note

All claims expressed in this article are solely those of the authors and do not necessarily represent those of their affiliated organizations, or those of the publisher, the editors and the reviewers. Any product that may be evaluated in this article, or claim that may be made by its manufacturer, is not guaranteed or endorsed by the publisher.

## Footnotes

^1^ https://www.wjx.cn/
