# Corrigendum: Work engagement and associated factors among healthcare professionals in the post-pandemic era: a cross-sectional study

**DOI:** 10.3389/fpubh.2023.1332486

**Published:** 2023-11-20

**Authors:** Yiya Wang, Li Tang, Lezhi Li

**Affiliations:** Clinical Nursing Teaching and Research Section, The Second Xiangya Hospital, Central South University, Changsha, Hunan, China

**Keywords:** work engagement, healthcare professionals, post-pandemic era, factors, cross-sectional study

In the published article, there was an error in affiliations 1, 2, 3. Instead of “^1^Xiangya School of Nursing, Central South University, Changsha, Hunan, China; ^2^Nursing Department, Henan Provincial People's Hospital, Zhengzhou, Henan, China; ^3^Clinical Nursing Teaching and Research Section, the Second Xiangya Hospital, Central South University, Changsha, Hunan, China”, it should be “Clinical Nursing Teaching and Research Section, the Second Xiangya Hospital, Central South University, Changsha, Hunan, China”.

The authors apologize for this error and state that this does not change the scientific conclusions of the article in any way. The original article has been updated.

