# Supplementary material for: Work engagement and associated factors among healthcare professionals in the post-pandemic era: a cross-sectional study
Source: Front Public Health. 2023 Jul 27;11:1173117. doi: 10.3389/fpubh.2023.1173117 (PMC10413104; doi:10.3389/fpubh.2023.1173117)
Supplement: Supplementary file 1 [file Table_1.docx]

Supplementary Material

Work engagement and associated factors among healthcare professionals in the post-pandemic era: A cross-sectional study

Yiya Wang^1†^, Li Tang^2†^, Lezhi Li^2*^,

*** Correspondence:** Lezhi Li: lilezhi@csu.edu.cn

**Supplementary table 1 The values of variables**

| Variables | Value |
| --- | --- |
| Gender | Male=1; Female=2 |
| Age | ＜25=1; 25~35=2; 36~45=3; 46~55=4; ＞55=5 |
| Marital status | Unmarried=1; Married=2; Others=3 (reference) |
| Occupation | Physician=1; Nurse=2; Others=3 (reference) |
| Professional title | Primary title=1; Intermediate title=2; Senior title=3 |
| Years of work experience | ≤5=1; 6-10=2; 11-15=3; 16-20=4; ≥21=5 |
| The severity of caring for patients with COVID-19: serious patients | No=0; Yes=1 |
| The severity of caring for patients with COVID-19: critical patients | No=0; Yes=1 |
| Whether having experience in the care of patients with COVID-19 | Yes=1; No=2 |
| Whether currently suffering challenges in the care of patients with COVID-19 | No=0; Yes=1 |
| Anxiety | negative=0; positive=1 |
| Depression | negative=0; positive=1 |
| Work-related basic need satisfaction | Original value |
| Workload | Original value |
| Self-kindness | Original value |
| Mindfulness | Original value |
